# A Systematic Review of the Immune-Regulating and Anticancer Activities of Pseudolaric Acid B

**DOI:** 10.3389/fphar.2017.00394

**Published:** 2017-06-28

**Authors:** Mei-lun Liu, Dan Sun, Tan Li, Hong Chen

**Affiliations:** ^1^Department of Pharmacognosy and Pharmaceutics, Logistics University of the Chinese People's Armed Police ForceTianjin, China; ^2^Department of Pathogen Biology and Immunology, Logistics University of the Chinese People's Armed Police ForceTianjin, China

**Keywords:** traditional chinese medicine, pseudolaric acid B, cancer, inflammation, immunoregulation

## Abstract

*Cortex pseudolaricis*, the root bark of Pseudolarix kaempferi Gord, has been used to treat tinea and other skin diseases for the antimicrobial activities in Traditional Chinese Medicine (TCM). Pseudolaric acid B (PAB) has been identified as the major component responsible for the action of *C. pseudolaricis*. Recently, PAB has been demonstrated to be used as novel treatments for cancer, immune disorders, inflammatory diseases, and immunosuppression. However, the mechanisms through which PAB exerts its properties are not understood well, and little attention in the literature has been given to review its pharmacological activities before. In this review, we performed a systematic summary of the literature with respect to the anticancer, immunosuppressive and anti-inflammatory properties of PAB and its derivatives. Currently available data suggest that PAB is a promising immunosuppressive and anti-inflammatory agent candidate and should be explored further in cancer treatment and prevention.

## Introduction

Pseudolaric acid B (PAB), a diterpene isolated from *Cortex pseudolaricis*, has been recorded as a common Traditional Chinese Medicine (TCM) to treat eczema, fungal skin infections, and other skin diseases for centuries. Previous studies indicated that PAB showed various bioactivities with less cytotoxicity, including antimicrobial activity, antifertility activity, antiangiogenic activity, anticancer activity, and so on (Wong et al., 2005; Liu et al., 2006; Ko et al., 2007; Yang et al., 2008; Yu et al., 2008; Li et al., 2009; Meng and Jiang, 2009). Especially as the further studies carrying out, PAB has been proved to markedly inhibit inflammatory reaction and regulate the immune response. The aforementioned properties suggest PAB is potentially of therapeutic value in the treatment of cancer, immune disorders, and other relevant diseases. Nevertheless, the mechanism responsible for PAB exerting the biological function is poorly understood. In view of this, the literatures about characteristic and correlative studies on PAB and its derivatives are reviewed which would provide the latest information and trend to further investigation PAB as a novel candidate against cancer and immune-related diseases.

## Source and structure

*C. pseudolaricis*, described as “tu-jin-pi” or “jin-qian-song-pi” in TCM, is the dried root bark of pseudolarix amabilis (Nelson) Regd. [Pseudolarix kaempferi Gord]. The crude medicinal ingredient contains pseudolaric acids, phenolic constituents, pigment and tannins (Li X. C. et al., 2014; Wu, 2015), among which PAB is the major bioactive extract and has been selected as its chemical marker to assess the quality of this herb.

**Figure 1:**
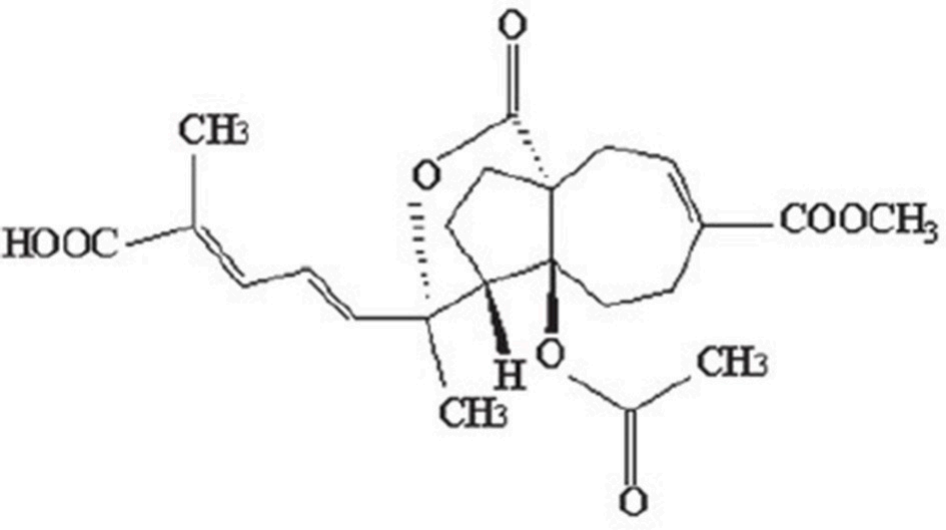
Chemical structure of PAB.

The monomer of PAB is a diterpene acid with a characteristic framework (Figure 1), which molecular formula is C_23_H_28_O_8_with a molecular weight of 432.464. The structure-activity relationship studies on PAB have revealed such distinctive functional groups in the molecule are crucial for the biological properties, as the *trans*-fused perhydroazulene framework bearing an ace-toxy/hydroxyl group and a lactone at the ring junction. Besides, the diene side chain appears to be important for activity (Chiu et al., 2010; Sarkar et al., 2012). To achieve significantly higher potency and minimal toxicity, additional studies on structural modifications for PAB are needed. Recently, the hydrogenated and indole derivatives of PAB have been considerably improved, and are more active for their anti-cancer and immunosuppressive properties than those of PAB, providing new insights for drug discovery and development (Li et al., 2014a; Yang et al., 2015; Zhao et al., 2016).

## Bioactivity

### Anticancer activity

Cancer could damage people's health and life seriously and has been a dangerous threat to human lifespan. Present therapeutics was mostly non-specific to aim directly at target tissues so that deleterious side effects occurred whereby the clinical application was restrained. Herein, researchers are devoting to develop new therapeutic strategy with higher safety and efficacy. There have been an increasing number of studies focusing on TCM to look for superior herbal candidates against cancer diseases.

It's well-known that TCM emphasizes the overall efficacy to treat cancer and inhibit cancer development and recurrence. So far, many Chinese herbs including PAB have been shown to have a good effect in cancer treatment, which display a broad range of clinical effects including alleviation of cancer-associated symptoms, prolonging survival rates, decreasing treatment-related toxicity, and preventing recurrence and metastasis. Although, the mechanism of PAB is still unclear, increasing evidence has shown that PAB has favorable anticancer effects through inhibiting cancer growth, regulating cell cycle, inducing apoptosis or autophagy, and so on, which covers gastric carcinoma, hepatocarcinoma, cervical cancer, breast carcinoma, lung carcinoma, melanoma, and so on (Yu, 2008; Sun and Li, 2014a). In this section, we will summarize the antagonistic effect and the molecular mechanisms of PAB on cancer cells according to recent researches.

#### The induction of apoptosis

Since targeting the apoptotic pathway and inducing apoptosis in cancer cells has been defined as a promising approach to the treatment of cancer, it is to be welcomed that some typical apoptotic morphology features such as cell shrinkage, cytoplasm vacuolization and apoptotic body has been observed in the morphological study of PAB-treated cancer cells (Xu et al., 2008; Hu et al., 2010; Zhao et al., 2012). There are some different pathways involved in the mechanisms of PAB to induce apoptosis including Bcl-2/Bax, caspases, ROS, JNK, ERK, p38, and NF-κB (Xu et al., 2015).

Bcl-2 family plays an important role in the modulation of apoptosis, composed of anti-apoptotic proteins, and pro-apoptotic proteins, among which Bcl-2 protein and Bax protein are, respectively the main representative. The ratio of Bcl-2/Bax takes on a higher level in cancer cells compared with the original cells (Um, 2016). Up-regulation of Bax and down-regulation of Bcl-2 could facilitate apoptosis in various cancer cells of different origins. Considering the significance of Bcl-2 family on apoptosis, Hu et al. have demonstrated that PAB showed dose-dependent inhibitory effect on Bcl-2 family in HeLa cells with the IC_50_ value of 10 μmol/L. After the treatment of 10 μmol/L PAB especially for 48 h, the mRNA expression of Bax enhanced and that of Bcl-2 decreased leading to the inhibition on proliferation and the apoptosis of HeLa cells (Hu et al., 2010).

The down-regulation of Bcl-2/Bax ratio has been also founded being accompanied by the activation of caspase family in the experiments on PAB-treated cancer cells (Xu et al., 2008; Yu et al., 2012; Yu B. et al., 2014; Zhao et al., 2012; Wang et al., 2013; Yao et al., 2016). Zhao et al. has reported about the anti-proliferative effect of PAB on DU145 cells, an *in vitro* model of hormone-refractory prostate cancer (HRPC) (Zhao et al., 2012). Photomicrographs of cells exposed to PAB presented typical apoptotic morphology with chromatin condensation and formation of apoptotic bodies, the appearance of which was regarded as the apoptotic hallmarks. Western-blot and caspase activity data indicated PAB down-regulated anti-apoptotic Bcl-2 protein and activated caspase-9 and caspase-3. The activation of caspases holds a material part in the apoptotic pathways. Caspase-3 is one of downstream effectors of the upstream initiators such as caspase-8 and caspase-9, which play a respective part in death receptor pathway and the mitochondrial pathway (Redza-Dutordoir and Averill-Bates, 2016). The experiment on MCF-7 cells, deficient of caspase-3, showed that 4 μmol/L PAB activated caspase-8 and caspase-9 to induce apoptosis. It's inferred that PAB induced MCF-7 cells apoptosis through both death receptor pathway and the mitochondrial pathway, bypassing caspase-3 (Tian et al., 2014). Furthermore, as the results of M Khan's experiment in U87 cells, z-VAD-fmk (the general caspase inhibitor) did not inhibit the apoptotic effect of PAB completely. This indicates that there is some caspase-independent apoptotic pathway involved (Khan et al., 2012).

In Zhao et al.'s study, a concentration-dependent increase in the percentage of ROS generation was also observed in DU145 cells after the treatment with PAB for 48 h. Such changes could be impeded by the pretreatment of NAC (a ROS scavenger) or MG-132 (an ubiquitin–proteasome blocker), suggesting PAB-induced apoptosis via ubiquitin–proteasome pathway-dependent ROS accumulation (Zhao et al., 2012). ROS was crucial for maintaining normal physiological functions during the development of cell cycle progression, proliferation and cell death (Um, 2016). PAB has evident effects on the increase of the level of ROS (Zhao et al., 2012; Liu et al., 2013; Qi et al., 2013a,b). Under PAB stimuli, excess cellular levels of ROS cause the Bcl-2 degradation and then trigger the cleavage of apoptotic caspases, finally leading to the activation of apoptosis. It is noteworthy that MAPK cascades are major intracellular signal transduction pathway, which are essential for the regulation of cellular development, such as survival and apoptosis. There are three main MAPK cascades, including the extracellular signal-regulated protein kinase cascade, the c-Jun N-terminal kinase (JNK) cascade, and the p38 MAPK cascade. In general, the activation of the JNK cascade and p38 MAPK cascade-mediated intracellular signaling would promote caspase-3 activation, which is indispensable for apoptotic morphological features (Mitomo et al., 2016). Based on the above analysis, Xu's et al. reported that early apoptotic cells increased time-dependently in SGC7901 cells after the exposure to PAB (Xu et al., 2008). The levels of phospho-JNK and phospho-p38 were attenuated by PAB, along with the increase of phospho-ERK, which indicated that PAB could affect MAPK pathway (Xu et al., 2008; Liu et al., 2013).

#### The inhibition of cell proliferation and regulation of cell cycle

Proliferation and apoptosis of cells are two critical factors that determine organism development and tissue homeostasis (Hervouet et al., 2013). Precisely, it is the unrestricted proliferation and suppressed apoptosis that make cancer cells grow frantically and malignantly. Accumulating studies have revealed that dysfunction in cell cycle regulation often resulted in abnormal proliferation of cancer cells (Hsu et al., 2013). In addition, both normal cells and cancer cells proliferate through mitosis and thus cell death often occurs during or after aberrant mitosis. Mitotic catastrophe could disturb cell cycle distribution and elicit senescence further leading to inhibit cell proliferation. Senescence is a process by which cells enter a state of permanent cell cycle arrest. Senescent cells are viable and retain their metabolic activity, although, they lose their proliferative capacity. PAB has been proved enable to obstruct mitosis and provoke cell cycle arrest in many cancer cells (Leander et al., 2008; Duan et al., 2010; Liu et al., 2012; Ma et al., 2012; Qi et al., 2012; Yu et al., 2013; Yao et al., 2014; Yu J. H. et al., 2015).

Duan et al. have found that PAB could block MCF-7 cells at G2/M phase based on DNA content distribution (Duan et al., 2010). The proportion of MCF-7 cells in G2/M phase rose up to 93% when the concentration of PAB was beyond 2.5 μmol/L. Further quantitive analysis to distinguish G2-phase cells and M-phase cells showed that PAB increased mitotic index (MI) obviously and promoted the expression of cyclin B1 in MCF-7 cells as well as high level of phospho-histone H3-positive cells (a hallmark of mitosis) suggesting that cell cycle arrest of MCF-7 cells induced by PAB mostly occurred in M-phase. Fluorescence staining illustrated that PAB could disrupt microtubules and interfere with the formation of mitotic bipolar spindles. Meanwhile, PAB induced grape-like giant nuclei and increased mitochondrial fluorescence intensity indicating mitotic slippage in MCF-7 cells. By this token, PAB exerted inhibitory effects on MCF-7 cell proliferation and induced apoptosis related to microtubule depolymerization.

There were similar results in murine fibrosarcoma L929 cells, which showed that PAB arrested the L929 cells in G2/M phase (76%) after 12~24-h exposure accompanied by the disruption of microtubule fibers (Qi et al., 2012; Yu et al., 2013). Phase contrast microscopy manifested that the PAB-treated cells initially exhibited a rounded morphology which was characteristic of mitotic cells and eventually died via apoptosis similar to the effect of colchicine. In fact, PAB caused the dysfunction of mitotic spindle apparatus and the absence of cytokinesis, which further inhibited microtubule polymerization and then resulted in multiple micronuclei. With respect to the molecular profile, PAB could up-regulate the positive regulator cyclin B1 and its accumulation potentially activated the cyclin B1-cdc2 complex. Meanwhile, PAB stimulated a dramatic increase in the phospho-histone H3 staining. Taken together, these data indicated that PAB induced L929 cells to an arrest in mitosis, but not in G2.

p53, a major mediator of cell cycle arrest, was reported to mutate or be inactivated in several cancers. If that happened, the apoptotic response is not activated and cell proliferation is allowed. Several studies have shown that p53 can up-regulate the level of p21 and Gadd45 to mediated cell cycle arrest (Mohammad et al., 2015). Previous studies have demonstrated that the expression of nuclear p53 was reduced by PAB treatment accompanied with the decreased senescence-associated β-galactosidase (SA-β-Gal) positive ratio (Khan et al., 2012; Qi et al., 2013b; Yao et al., 2014, 2016; Yu J. H. et al., 2015). The suppression of p53 and p21 was associated with decreased senescence induction in the PAB-treated A549 cells and H460 cells. In addition, p53 down-regulation reduced the protein levels of p53, p-p53, and p21. Consistently, the knockdown of p21 attenuated PAB-induced senescence. These results suggest that PAB-induced senescence might require functional p53 (Yao et al., 2016).

However, in some cases, mitotic catastrophe (MC) means a survival mechanism for cancer cells. As is known, both ATM-Chk2-cdc25 and ATM-p53-p21 signal pathways could mediate the cell cycle arrest following DNA damage (Leander et al., 2008; Ma et al., 2012). In PAB-treated HeLa cells at a low concentration, the expression of these pathways-related proteins increased, consequently leading to G2/M phase arrest. The treatment with ATM inhibitor or specific inhibitor of p53 augmented the growth inhibitory ratio of PAB-treated HeLa cells, when apoptosis changed to be protection for PAB-treated HeLa cells survival instead (Yao et al., 2014).

#### Autophagy-dependent senescence

Programmed cell-death may be achieved through two distinct processes, which contains apoptosis and autophagy. Autophagy, which may contribute to cell death or cell survival, is characterized by the appearance of autophagosomes that engulf bulk cytoplasm and cytosolic organelles, such as mito-chondria and endoplasmic reticulum. Lysosomes fuse with the autophagic vesicles, leading to degradation of their cargo. Autophagy recycles cellular material for survival, however, its continuation leads to organelle degradation and ultimately cell death (Liu et al., 2016).

The lipidated form of microtubule-associated protein-1 light chain 3 (LC3II) is generated during autophagosome formation, the amount of which is a good indicator of autophagosome formation. It is well documented PAB could increase the conversion from LC3I to LC3II and the positive ratio of MDC staining which marked autophagy without subdiploid peak of apoptosis marker (Qi et al., 2013a,b; Tong et al., 2013; Yu et al., 2013; Yu J. et al., 2016; Yu J. H. et al., 2015). 3MA inhibits class III phosphoinositide 3-kinase (PI3K) and is widely used as an inhibitor of autophagy in mammalian cells (Mizushima et al., 2010). Further studies manifested that 3MA treatment obviously reduced the ratio of SA-β-Gal-positive cells and the p21 protein levels indicating that autophagy facilitated senescence in PAB-treated L929 cells. And it covered approximately 53% of the cells suffering from mitotic catastrophe. Additionally, p19-p53-p21 and p16-Rb pathways are involved in the senescence process as well as the reduction on AKT/mTOR activity (Qi et al., 2012, 2013b). ROS is sufficient to trigger mitochondrial dysfunction (Skulachev, 2009). Based on Qi et al.'s data, both ROS and mitochondrial dysfunction were evidently induced by PAB in L929 cells. They have also found that NAC significantly decreased the MDC-positive ratios and the conversion from LC3I to LC3II. Besides, 3MA or CQ (a lysosomal inhibitor) effectively reduced intracellular ROS generation and mitochondrial superoxide production in PAB-treated cells. According to all these results, Qi et al. concluded that a positive feedback loop between autophagy and ROS/mitochondrial dysfunction existed in PAB-treated L929 cells (Qi et al., 2013a,b).

#### The inhibition of angiogenesis

It is well-established that angiogenesis is necessary for the cancer progression and metastasis (Qi et al., 2015). The inhibition of cancer angiogenesis has therefore become an important strategy for cancer therapy. Angiogenesis occurs by basement membrane degradation by proteases, endothelial cell proliferation and migration/invasion, formation of capillary tubes, survival of newly formed blood vessels and so on, which is tightly regulated by an intricate balance between stimulators and inhibitors such as Vascular Endothelial Growth Factor (VEGF), fibroblast growth factor, and matrix metalloproteinases (MMPs) (Hseu et al., 2011). VEGF is an essential activator involved in the pathologic link between angiogenesis and cancer growth. As a highly specific endothelial cell mitogen, VEGF can promote endothelial cell proliferation, migration, and survival, resulting in cancer blood vessel formation (Toomey et al., 2009).

Endothelial cell movement is a critical step during cancer-induced angiogenesis, which is the foundation of neovascularization and cancer metastasis. The statistical data in the cell motility assay indicated that PAB inhibited the migratory potential of gastric cancer cells and human umbilical vein endothelial cells (HUVECs) (Liu et al., 2012; Yu et al., 2012). Microvessel density (MVD) is an important indicator for measuring cancer angiogenesis. It is often assessed by determining the expression levels of CD31 staining (Kumar et al., 2009). The results from Liu et al.'s studies indicated that the value of MVD in the PAB group was approximately half of that in the control group. PAB inhibited angiogenesis through the down-regulation of VEGF gene at the transcription and translation levels, which caused a reduction of CD31 expression in blood vessels (Liu et al., 2012).

Besides, hypoxia inducible factor 1α (HIF-1α) is a transcription factor that drives neoangiogenesis by regulating the expression of various target genes, including VEGF and VEGFR, in response to hypoxia during the growth of solid tumors. It's evident that PAB could down-regulate the levels of mRNA and protein expression of HIF-1α (Liu et al., 2012; Miao et al., 2012; Yu et al., 2012; Wang et al., 2017). Li et al. discovered that it occurred because PAB induced the proteasome-executed degradation of the HIF-1α protein (Li et al., 2004). Further studies have confirmed that PAB accelerated the phosphorylation of c-Jun at Ser63/73 executing a non-transcriptional function to stabilize HIF-1α (Yu et al., 2012).

#### The reversal of multidrug resistance

Although, chemotherapy is the main strategy for the treatment of cancer, a major problem limiting its success is the intrinsic or acquired drug resistance. Therefore, multidrug resistance is a major impediment in medical oncology resulting in a failure of a successful cancer treatment (Büsselberg and Florea, 2017). It has been discovered that the major reason for MDR in cancer is the overexpression of P-glycoprotein (P-gp), a product of the human MDR1 gene. Besides, recent studies have demonstrated that in cancer cells with MDR, the expression levels of Cyclo-oxygenase-2 (Cox-2) and protein kinase C-a (PKC-α) are both up-regulated, and their inhibition can reverse neoplastic MDR. An experiment in a P-gp-overexpressing cell line (SGC7901/ADR) demonstrated that PAB significantly suppressed the tumor growth. The combination of PAB and the traditional chemotherapy drug Adriamycin (ADR) exhibited more potent inhibitory effects on the growth of gastric cancer *in vivo* than treatment with either PAB or ADR alone (Yu F. et al., 2015). Especially, the higher expression of Cox-2, PKC-α, and P-gp in SGC7901/ADR cells was weakened after the combination treatment of PAB and ADR(Sun and Li, 2014b; Yu F. et al., 2015). This manifested that PAB could reverse the multidrug resistance to cancer cells via down-regulating Cox-2-PKC-α-P-gp/MDR1 signal pathway (Wong et al., 2005; Liu et al., 2012; Sun and Li, 2014b; Yu F. et al., 2015).

### Anti-inflammatory activity

Numerous experiments have proved that natural terpenoids could exert inhibition effects on various inflammatory models to different degrees (Hou et al., 2015). “Tu-jin-pi” has been widely used to treat microbial skin diseases for centuries. Relative studies indicated PAB had indeed strikingly anti-inflammatory activities. In the 2, 4-dinitrofluorobenzene (DNFB)-induced delayed-type hypersensitivity (DTH) mouse model, the ear thickness and ear weight of the mice were obviously reduced by the application of PAB dose-dependently with diminished thymus and spleen indexes, showing a suppression of inflammatory infiltration and dermal edema, as the same to Hexahydropseudolaric acid B (HPAB), a novel hydrogenated derivative of PAB, which showed more favorable immunomodulatory effect (Li et al., 2012, 2013, 2014a,b; Li T. et al., 2015; Yang et al., 2015; Hu et al., 2016). Although, such researches have extended the understanding of PAB on the anti-inflammatory and immunosuppressive effects, the certain underlying molecular mechanism requires further exploration. In this section, we mainly introduce the recorded anti-inflammatory and immunoregulatory effects of PAB.

#### The effect on inflammatory cytokines

Inflammatory reaction stimulates vascular permeability and white blood cells infiltrate causing tissues injury during which a large number of inflammatory cytokines are implicated in. Li et al. observed a significant decline for the expression of IFN-γ and IL-6 in the murine contact hypersensitivity model (CHS) administrated with PAB (Li et al., 2012). In contrast, topical application of PAB contributed to increase the mRNA expression of IL-4, IL-10, and TGF-β and down-regulate IFN-γ, IL-6, IL-17, and TNF-α (Li et al., 2012, 2016; Li Y. X. et al., 2015).

Since macrophage is of significance in the inflammatory reaction with functional versatility and plasticity, macrophages phenotype polarization will be activated under certain inflammatory conditions and thus result in the changes of corresponding cytokines expression. The classically activated macrophage (M1 phenotype) secretes inflammatory-stimulating cytokines for example TNF-α, IL-1β, IL-6, COX-2, iNOS, and PGE2, and then promotes the inflammatory response; the alternatively activated macrophage (M2 phenotype) has the opposite effect of suppressing inflammatory (Salvi et al., 2017).

After LPS-stimulation, RAW264.7 macrophages deviate to the M1 phenotype rather than the M2 phenotype. Li et al. reported that PAB significantly reduced the mRNA levels of M1 phenotype markers (IL-1β, iNOS, TNF-α), while raised the mRNA levels of M2 phenotype markers (Arg1, Mrc1) instead indicating that PAB have an inhibitory effect on the M1 phenotype polarization (Li et al., 2012, 2016; Li Y. X. et al., 2015). In summary, PAB has notable influence on the correlative inflammatory cytokines to restrain inflammation (Li et al., 2012, 2016; Li T. et al., 2015; Li Y. X. et al., 2015; Hu et al., 2016).

#### The regulation on signal pathways

It's well-known that a variety of signal pathways transmitting correlative messages are implicated in the processes of inflammatory response and the production of inflammatory cytokines. Thus, this part focused on the signal pathways associated to the anti-inflammatory activity of PAB. PAB could exert positive effects on the p38 MAPK signal cascades, and the downstream proteins of p38 MAPK pathway Activated p38 MAPK could trigger the phosphorylation of MK2 and HSP27 directly or indirectly. Consistent with the decreasing level of phospho-p38 MAPK, the reduction of p-MK2 and p-HSP27 was presented after treatment with PAB (Li et al., 2014a; Li T. et al., 2015). Meanwhile, the activation of p38 MAPK was also inhibited by HPAB in DNFB-induced DTH mice (Li et al., 2014a). In addition, the amount of p65 and phospho-p65 (ser276), which was often detected for NF-κB pathway, underwent a low level after PAB stimulation (Li et al., 2009). The phosphorylation and degradation of IκBα, known as an inhibitor protein of NF-κB, showed a dose-dependent suppression at PAB concentrations of 1–10 μmol/L (Wei et al., 2013). It was concluded that NF-κB pathway was involved in the mechanism responsible for PAB inhibition effects on inflammation (Li et al., 2009, 2016).

Peroxisome proliferator-activated receptors (PPARs) are a group of nuclear receptor proteins that play an essential role in the LPS-induced inflammatory reaction (Yin et al., 2014). It has been investigated that PAB could function as an agonist of PPAR-γ via stimulating PPAR-dependent gene transcriptions (Li et al., 2012, 2014a,b, 2016; Li T. et al., 2015).

### Immunosuppressive activity

According to the pharmacology researches, many natural medicines could regulate the immune response through exerting the anti-inflammatory activities. Recently, increasing attention has been paid to the immunomodulatory properties of PAB.

#### The inhibition of the lymphocytes proliferation

The activation of the immune response is often characterized by the proliferation of T and B lymphocytes. Previous studies have pointed out the effects of PAB on the proliferation of lymphocytes (Li et al., 2009, 2012, 2013, 2014b; Wei et al., 2013; Chen et al., 2015; Li T. et al., 2015).

Wei et al. investigated the immunosuppressive activity of PAB upon the proliferation of T lymphocytes and B lymphocytes stimulated by concanavalin A (Con A) and lipopolysaccharide (LPS), respectively, the proliferation of antigen-specific T lymphocytes evoked by dinitrophenyl-modified spleen cell (DNP-SC) was also measured (Wei et al., 2013). The founding showed that PAB suppressed antigen-specific T lymphocytes proliferation (DNP-SC-evoked) significantly and exerted a higher inhibitory effect on the proliferation of T lymphocytes (Con A-induced) than that of ciclosporin A (CsA). However, B lymphocytes proliferation (LPS-induced) was not impacted dramatically. Meanwhile, the production of IL-2, one of the primary cytokines released by T-cell activation, declined obviously following PAB-treatment.

Moreover, HPAB turned out to be more preferably immunosuppressive than PAB, which significantly inhibited the proliferation of T cells stimulated by Con A dose-dependently with higher IC50 values than those of PAB (Li et al., 2014a; Yang et al., 2015). In agreement with above-describes, PAB could be effective to suppress the proliferation of spleen-lymphocytes from DTH mice as well as antigen-specific T lymphocytes *in vitro* (Li et al., 2013).

#### The effects on CD4^+^ T cells

Naive CD4^+^ T cells could differentiate into Th1, Th2, Th17 cells and CD4^+^CD25^+^Tregs while responding to the character of the threat encountered and then, respectively secrete their signature cytokines (O'Shea and Paul, 2010).

Th1 cells could secrete cytokines including IL-2, IFN-γ, TNF-β and others, and express nuclear factors dominantly like T-bet so that Th1 cells facilitate the process of macrophage-mediated immune response. Th2 cells could stimulate the proliferation of B lymphocytes and boost the humoral immune-response with the production of IL-4, IL-5, IL-13 and the expression of GATA-3, a Th2-specific transcription factor. CHS is a form of DTH reaction generally accompanied by the Th1 cells-mediated disorder. It's well-documented that PAB could interfered with Th1 response on the CHS mice, as the CD4^+^IFN-γ^+^ cells (Th1) markedly reduced in a dose-dependent manner and the ratio of T-bet/GATA-3 decreased (Li et al., 2012).

What's more, recent studies demonstrated that the balance between Tregs and Th17 cells also could be affected by the administration of PAB. Tregs/Th17 balance is necessary for the inflammatory and immune-associated diseases (Liu et al., 2016; Fasching et al., 2017), among which Tregs could produce the regulative cytokines, IL-10 and TGF-β, and preferentially express forkhead transcriptional factor, Foxp3. Whereas Th17 cells secret mainly IL-17, IL-21, and other pro-inflammatory cytokines. The hallmark transcription factor, RORγt is of great significance during the differentiation of Th17 cells, which mainly secret IL-17, IL-21, and other pro-inflammatory cytokines.

From the lower expression levels of IL-17, RORγt and the enhancement of Foxp3 in the draining LNs from PAB-treated CHS mice, the development of Th17 cells proved to be inhibited with providing a negative-feedback loop and thus suppressed the activation of immune response. On the other hand, the obvious increase in the proportion of CD4^+^CD25^+^Foxp3^+^ cells (Tregs) and the higher gene expression of IL-10 and TGF-β in the CHS mice with PAB-application suggested the generation of Tregs including both nTregs and tires was promoted (Li et al., 2012). The similar result was occurred in the DTH mice that the level of CD4^+^CD25^+^Foxp3^+^ Tregs elevated from 2.5% up to a near-normal level of 2.9% after the treatment with 0.1% PAB (Li et al., 2013). Similarly, 15 mg/kg HPAB preferably promoted the percentage of CD4^+^CD25^+^Foxp3^+^ Tregs to 2.2% higher than the normal level along with the increasing expression of Foxp3, TGF-β, and IL-10 (Li et al., 2014a).

Further, Li et al. demonstrated PAB modulated the Tregs/Th17 balance via suppressing IL-6 expression through STAT3 pathway (Li et al., 2012). HPAB induced Tregs accompanied by the high expression of p-AKT and the reduction of phospho-p38 MAPK as well as the downstream proteins, so that it's supposed the underlying mechanism would be related to the inhibition of p38 MAPK signal cascades and AKT activation (Li et al., 2014a). Additionally, the enhancement of PAB and HPAB on PPAR-γ expression and the reversal effect of GW9662, a PPAR-γ antagonist, suggested PPAR-γ was involved in the promotion on Tregs for PAB and HPAB (Li et al., 2012, 2014a; Li T. et al., 2015). Thus, PAB could be regarded as a novel immunosuppressive agent through regulating Th1/Th2 and Th17/Treg balance.

## Conclusions and prospect

**Figure 2:**
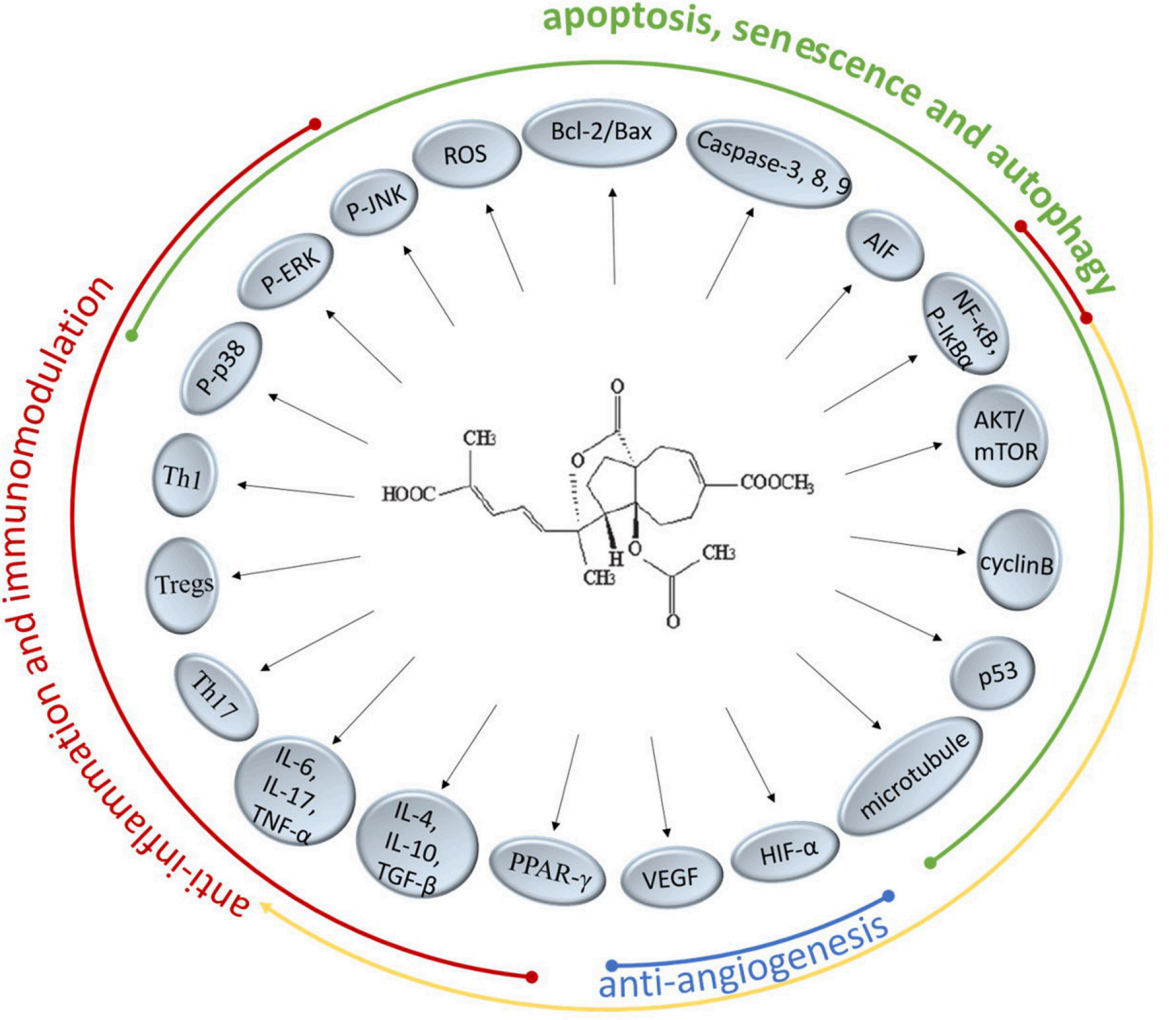
Molecular targets and pathways of PAB.

There are extensive anticancer drugs and immune-suppressants with powerful therapeutical effects, however, their adverse reaction shouldn't be overlooked whereby the clinical application is restrained. PAB has been proved to markedly inhibit many kinds of cancer cell lines and regulate the immune responses especially with less cytotoxicity. In the present researches on TCM, it's been found that PAB could intervene numerous molecular targets and pathways to achieve performance function (Figure 2). It might offer a potential access to a novel agent for treating cancer or immunological diseases.

It's well-known that Bcl-2 protects many cell lines from induced apoptosis by forming heterodimer with Bax. PAB have the certain effects on regulating the Bcl-2/Bax ratio via intracellular ROS accumulation and could trigger caspase-dependent apoptosis in death receptor pathway and the mitochondrial pathway, the precise mechanism of which is a subject remaining to be researched. PAB also suppressed the COX-2 protein expression and significant nuclear translocation of NF-κB and STAT3 (Hou et al., 2012). Besides, the increased expression of apoptosis inducing factor (AIF) directly inducing DNA fragmentation indicated that caspase-independent apoptotic pathways are also involved (Khan et al., 2012). PAB could not only induce the apoptosis of cancer cells but also inhibit the proliferation through blocking cancer cells at G2/M phase and provoking cell senescence and autophagy, which is related to the anti-microtubule activity and the regulatory function for p53 of PAB. Qi et al. pointed out PAB-induced autophagy-dependent senescence was only observed in some specific cell lines but not in all. Further understanding how autophagy and senescence function cooperatively in the process of PAB exerting inhibition effect need to be elucidated. Previous reports have confirmed VEGF and HIF-1α were targets of PAB supressing neoangiogenesis. A cancer grows rapidly and nearly exponentially once vascularized, therefore, inhibition of cancer angiogenesis has been one of the promising activity for PAB in the development of a novel anticancer candidate. Since multidrug resistance (MDR) limited the success of therapy in patients treated long-term with chemotherapeutic drugs, it's welcome that PAB displayed obvious inhibitory effects toward drug-resistant cancer cells. But the underlying mechanisms of PAB reversing MDR require more concrete descriptions in future studies.

Recent pharmacological studies have showed PAB and its derivatives could selectively suppress T lymphocytes *in vitro* and antigen-specific T lymphocyte proliferation, which confirms the suppressive activity of PAB on T lymphocytes. Moreover, the preliminary results suggested PAB could modulate the balance of the differentiated CD4^+^ T cells (Th1, Th17, and Tregs) and then affect the corresponding cytokines to exert the immunomodulatory properties. On the other hand, PAB could inhibit the mRNA and protein levels of inflammatory cytokines to reduce inflammation, which is associated with the p38-MAPK pathway, NF-κB pathway and PPAR-dependent gene transcriptions. More in-depth and concrete studies are needed to uncover the mechanisms of anti-inflammation and immunomodulation activity of PAB.

Although, discussion remains about whether or not PAB and its derivatives can be administered for cancer and immune-related diseases, they are an interesting new treatment option and further research in the area should be encouraged.

## Author contributions

ML was responsible for analyzing the relevant materials and wrote the manuscript; DS helped to search and collect the literatures on the Pseudolaric acid B; TL and HC handled the work of revising the manuscript.

### Conflict of interest statement

The authors declare that the research was conducted in the absence of any commercial or financial relationships that could be construed as a potential conflict of interest.
